# Lipopolysaccharide Exposure Differentially Alters Plasma and Brain Inflammatory Markers in Adult Male and Female Rats

**DOI:** 10.3390/brainsci12080972

**Published:** 2022-07-24

**Authors:** Hannah A. Nonoguchi, Timothy Wee Shang Kouo, Sandhya Kortagere, Joshua Hillman, David L. Boyle, Chitra D. Mandyam

**Affiliations:** 1 VA San Diego Healthcare System, San Diego, CA 92161, USA; hnonoguc@ucsd.edu (H.A.N.); twkouo@ucsd.edu (T.W.S.K.); 2 Department of Microbiology & Immunology, College of Medicine, Drexel University, Philadelphia, PA 19104, USA; sk673@drexel.edu; 3 Department of Medicine, University of California San Diego, San Diego, CA 92093, USA; jhillman@health.ucsd.edu (J.H.); dboyle@health.ucsd.edu (D.L.B.); 4 Department of Anesthesiology, University of California San Diego, San Diego, CA 92093, USA

**Keywords:** LPS, meso scale discovery, prefrontal cortex, dorsal striatum, hippocampus, sex differences

## Abstract

Humans and rodents have sexually dimorphic immune responses, which could influence the brain’s response to a systemic inflammatory insult. Lipopolysaccharide (LPS) is a stimulator of the innate immune system and is routinely used in animal models to study blood–brain barrier (BBB) dysfunction under inflammatory conditions. Therefore, we examined whether inflammatory response to LPS and the associated BBB disruption differed in male and female adult rats. Rats were treated with saline or two injections of 1 mg/kg LPS and studied 24 h after the second LPS injection. Plasma isolated from trunk blood and brain tissue homogenates of the prefrontal cortex (PFC), dorsal striatum (DS), hippocampus, and cerebellum were analyzed for cytokines and chemokines using a 9-plex panel from Meso Scale Discovery. BBB disruption was analyzed with tight junction proteins claudin-5 and VE-cadherin via Western blotting and VEGF by ELISA. This allowed us to compare sex differences in the levels of individual cytokines as well as associations among cytokines and expression of tight junction proteins between the plasma and specific brain regions. Higher levels of interferon-γ, interleukin-10 (IL-10), IL-13, IL-4, CXCL-1, and VEGF in the plasma were revealed compared to the brain homogenates, and higher levels of TNFα, IL-1β, IL-6, and IL-5 in the PFC were seen compared with plasma and other brain regions in males. Females showed higher levels of plasma CXCL1 and VEGF compared to males, and males showed higher levels of PFC TNFα, IL-6, IL-4, and VEGF compared to females. LPS induced significant increases in plasma cytokines and VEGF in both sexes. LPS did not significantly alter cytokines in brain tissue homogenates, however, it increased chemokines in the PFC, DS, and hippocampus. In the PFC, LPS produced BBB disruption, which is evident as reduced expression of claudin-5 in males and reduced expression of VE-cadherin in both sexes. Taken together, our results reveal significant sex differences in pro-inflammatory cytokine and chemokine levels in plasma and brain that were associated with BBB disruption after LPS, and validate the use of multiplex assay for plasma and brain tissue samples.

## 1. Introduction

Sickness behavior mediated by acute cytokine challenge and psychological disorders in response to chronic neuroimmune responses have been reported in humans and animals [1,2,3,4,5]. For example, lipopolysaccharide (LPS) exposure in rodents induces a host of sickness, anxiety-like and depression-like behaviors, partly attributed to the central and peripheral induction of inflammatory cytokines [6,7]. Given the limited studies on the sex-specific effects of LPS on the expression of cytokines, chemokines, and leukocytes in the brain and periphery, and the significant influence of sex on neuroimmune responses [8,9,10,11], it is unclear whether the periphery and the brain regions implicated in the neurobiology of learning and memory, anxiety, depression, and negative affect differentially respond to peripheral immune challenges in a sex-specific manner.

The multifactorial aspects of neuroimmune communication were elegantly reviewed recently [12]. For example, there is significant data supporting the associations between a number of immune mediators, including inflammatory cytokines, and the pathogenesis of neuropsychiatric disorders, such as depression, anxiety, and schizophrenia [1]. In order to understand the relationship between brain and immune system, several studies have relied on systemic immune activation using bacterial components such as LPS, to report that immune mediators, such as cytokines, can have a significant effect on brain function and behavior [8,9,11,13]. In addition to stimulating neuronal circuits, systemic immune activation-mediated pro-inflammatory cytokines can stimulate the blood–brain barrier (BBB), perivascular macrophages, or microglia within circumventricular organs, and therefore recruit leukocytes into the brain [12]. It has been hypothesized that this phenomenon could be driven by chemokines and could contribute to an understudied route of neuroimmune responses [13]. 

Prenatal, neonatal, and middle-age LPS treatment in rodents indicated infiltration of immune cells into the brain parenchyma, which coincided with increases in levels of cytokines and chemokines, in the prefrontal cortex (PFC), striatum, and hippocampus [8,9,10]. In parallel, LPS treatment in these age groups also produces learning and memory deficits dependent on the cortex and hippocampus, suggesting that altered cytokine and chemokine levels in the brain may assist with these deficits. LPS treatment during adulthood also increases whole brain cytokine levels, however, the alterations in specific brain regions implicated in the neurobiology of learning and memory, anxiety, depression, and negative affect is unknown [11,14]. Notably, studies in humans and rodents have indicated that sex can be linked to immune differences [8,9,10,11,15,16], with females responding more robustly to immune challenges, centrally and peripherally, compared to males. However, the effect of sex on immune responses, particularly pro-inflammatory responses, in brain regions implicated in neurobiology of learning and memory, anxiety, depression, and negative affect in adult rodents remains unclear. Using unbiased approaches, we tested the hypothesis that a peripheral immune challenge would produce higher levels of pro-inflammatory cytokines and chemokines, particularly in the brain regions associated with learning and memory, anxiety, depression, and negative affect (PFC, striatum, hippocampus; [17,18]) in female rats compared with males. We also investigated a sub hypothesis that the exaggerated pro-inflammatory responses in the PFC would be associated with greater dysfunction of the BBB in female rats compared to males.

## 2. Materials and Methods

### 2.1. Animals

Experimental procedures were carried out in strict adherence to the National Institutes of Health Guide for the Care and Use of Laboratory Animals and approved by the Institutional Animal Care and Use Committee of VA San Diego Healthcare System (protocol # A16-000). A total of seven adult males (weighing 300–350 g at the time of the experiment) and nine adult females (weighing 180 to 220 g at the time of the experiment) 10-week-old Long-Evans rats (bred at the VA Vivarium), were housed two per cage in a temperature-controlled vivarium under a reverse light/dark cycle (lights off 9:00 a.m.–9:00 p.m.) until completion of the study.

### 2.2. LPS Injections

**Figure 1:**
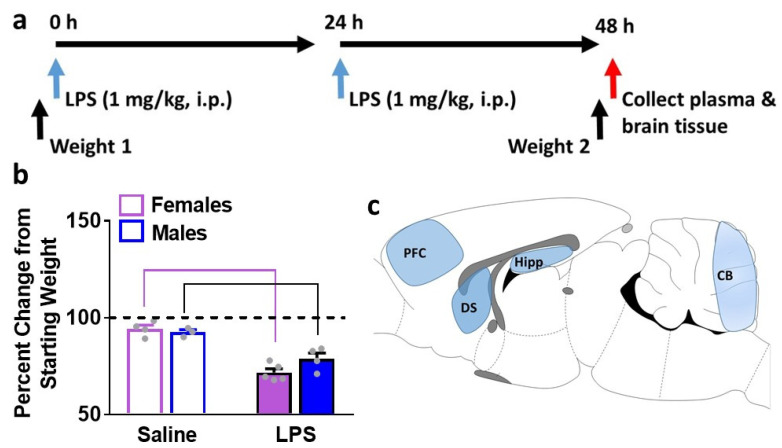
Schematic of the time line of body weight measures, LPS injections, and euthanasia (**a**). Body weight data expressed as percent change in weight 2 from weight 1 (**b**). Sagittal view of the adult rat brain indicating the region of interest for dissections (**c**). Prefrontal cortex, PFC; dorsal striatum, DS; hippocampus, Hipp; cerebellum, CB. Data are represented as mean ± S.E.M. n = 4 control female, n = 5 LPS female, n = 3 control male, n = 4 LPS male. *p* < 0.05 vs. controls by post-hoc tests.

Adult female (n = 5) and male (n = 4) Long-Evans rats received intraperitoneal injections of 1.0 mg/kg LPS from escherichia coli (Sigma-Aldrich, St. Louis, MO, USA, L2630) in sterile saline (0.9%) once per day for 2 consecutive days (Figure 1a). Rats were euthanized 24 h after the second injection of LPS. The injection paradigm was designed based on a prior report in adult rats, where this paradigm at the 24 h time point produced activated microglia in the brain [19]. This LPS injection paradigm in mice also produced moderate sickness behavior and transient weight loss, in parallel with increases in plasma and brain cytokines and activated microglia in the brain when detected 3 h after the second LPS injection [6]. Since weight loss was not measured at the 24 h time point, and since measures of peripheral and central immune factors (cytokines, chemokines and VEGF) have not been evaluated at this time point, we chose this time point in our study. Control rats (n = 4 females and n = 3 males), age matched, received saline injections in the same paradigm. Control rats were euthanized 24 h after the second injection of saline.

### 2.3. Termination, Plasma and Tissue Collection

Rats were quickly decapitated under isoflurane anesthesia, and trunk blood was collected in heparinized tubes and centrifuged at 4000 rpm for 5 min at 4 °C with supernatant plasma collected for total protein quantification and cytokine analysis. Brain tissue was immediately dissected on ice and regions including the prefrontal cortex (PFC), dorsal striatum (DS), entire hippocampus (Hipp) and cerebellum (CB; Figure 1c) were placed into lysis buffer (50 mM Tris, 1 mM EDTA, 320 mM sucrose, 1 mM PMSF) containing phosphatase and protease inhibitors (Sigma) and immediately homogenized in a refrigerated bead mill homogenizer (Next Advance). Following homogenization, all tissue samples were centrifuged at 6000 rpm for 5 min at 4 °C with supernatant collected for total protein quantification and cytokine analysis. Protein concentration was determined by a detergent-compatible Lowry method (Bio-Rad, Hercules, CA, USA). Samples were stored at −80 °C.

### 2.4. Multiplex Cytokine Immunoassays

Cytokine levels in plasma and brain tissue homogenates were analyzed using a custom Meso Scale Discovery rat cytokine v-plex panel, allowing for the measurement of interferons (IFN-γ), interleukins (IL-1β, IL-4, IL-5, IL-6, IL-10, IL-13), chemokines (KC/GRO (CXCL1)), adipokines (TNF-α; (catalog #: K15059D, MSD, Rockville, MD, USA)), and VEGF r-plex, allowing for the measurement of rat VEGF-A (catalog #: F231F). Plasma samples were diluted by adding 30 µL plasma to 90 µL of diluent provided with the kit and 50 µL of the diluted plasma was used per well. Brain protein extracts were normalized to 1 µg/µL and diluted by adding 10 µL of protein extract to 40 µL of diluent per well. Plates were read with a Sector Imager 2400, and data analyzed using the MSD Discovery Workbench software v. 4.0 (MSD, Rockville, MD, USA). The lower limit of detection (LLOD) for the assays varied by analyte. The following are LLOD for each marker (pg/mL): IFN-γ: 0.65, IL-1β: 6.92, IL-4: 0.69, IL-5: 14.1, IL-6: 13.8, IL-10: 16.4, IL-13: 1.97, KC/GRO (CXCL1): 1.04, TNF-α: 0.72, and VEGF: 0.69. Values falling below the LLOD were replaced with 0 pg/mL in all analyses and figures. IL-5 was not detected in plasma. 

### 2.5. Western Blot Analysis

PFC tissue lysates were mixed (1:1) with a Laemmli sample buffer containing β-mercaptoethanol. Each sample containing protein from one animal was run (30 μg per lane) on 10% SDS-PAGE gels (Bio-Rad, Hercules, CA, USA) and transferred to polyvinylidene fluoride membranes (PVDF pore size 0.2 μm). Blots were blocked with 5% milk (*w*/*v*) in TBS (25 mM Tris−HCl (pH 7.4), 150 mM NaCl) for 1 h at room temperature and were incubated with the primary antibody for 16–20 h at 4 °C: Antibody to phosphorylated-NFkB-p65 at Ser536 (rabbit polyclonal, 1:200, Cell Signaling cat# 3033L, molecular weight 65 kDa); total NFkB-p65 (rabbit polyclonal, 1:500, Cell Signaling cat# 8242S, molecular weight 65 kDa); Cox-2 (rabbit monoclonal, 1:500, Cell Signaling cat# 12282S, molecular weight 75 kDa); VE-cadherin (mouse monoclonal, 1:100, Santa Cruz biotechnology cat# sc-52751, molecular weight 60 kDa); and Claudin-5 (mouse monoclonal, 1:500, Invitrogen cat# 35-2500, molecular weight 20 kDa). Blots were then washed three times in TBS Tween20 (0.1%) and incubated for 1 h at room temperature with horseradish peroxide–conjugated goat antibody to mouse or rabbit in 2.5% milk in TBS. Following subsequent washes, immunoreactivity was detected using Supersignalwest Dura chemiluminescence detection reagent (Thermo Scientific, Waltham, MA, USA) and images were collected using a digital imaging system (Azure Imager c600, VWR, Radnor, PA, USA). For normalization purposes, membranes were incubated with 0.125% coomassie stain for 1–2 min and washed three times for 5–10 min in destain solution [20,21]. Densitometry was performed using ImageJ software (NIH). For total proteins, the signal value of the band of interest is expressed as a ratio of the corresponding coomassie signal. For phosphoproteins, the signal value of the band of interest is expressed as a ratio of the corresponding total protein signal. This ratio of expression for each band is then expressed as a percent of the control included on the same blot.

### 2.6. Statistical Analyses

Effects of LPS on cytokine levels in plasma and brain lysates in female and male rats were analyzed using a two-way ANOVA. Effects of LPS on protein levels with Western blot analysis were analyzed with unpaired t test using GraphPad Prism version 7. Tukey’s post-hoc analyses were conducted when a significant interaction was detected. Significance was set at *p* < 0.05.

## 3. Results

### 3.1. LPS Treatment Reduces Body Weight in Female and Male Adult Rats

A previously published model of LPS exposure was used in female and male rats [6,19]. This triggered a significant drop in body weight in both sexes (Figure 1b). Saline injections in females reduced body weight by 5 ± 1.9%, and in males by 7 ± 1.3%, and data was not significant. LPS injections in females significantly reduced body weight by 28 ± 1.9%, and in males by 21 ± 2.9% (*p* < 0.05).

### 3.2. Levels of Cytokines, Chemokines and VEGF in Healthy Control Rats: Sex Differences in Blood and Brain Homogenates

**Figure 2:**
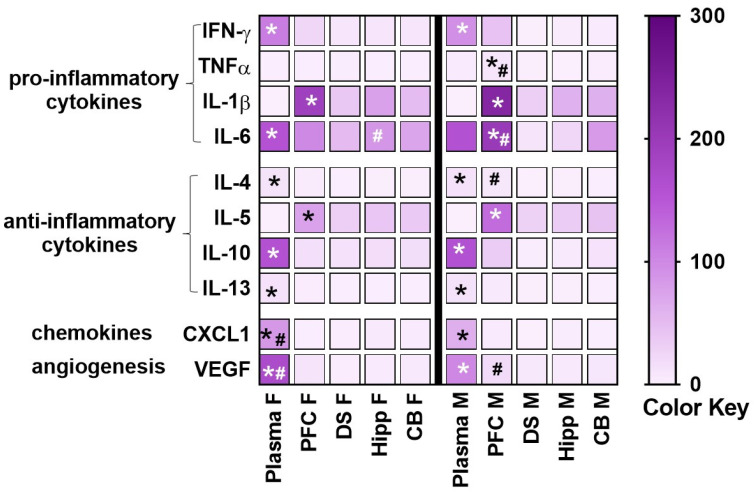
A heatmap was generated to display 10 different measured inflammatory markers in healthy control (saline treated) female (F) and male (M) rats in plasma and brain tissue homogenates. Mean values without standard error are indicated in the heatmap. However, statistical analysis was performed on mean ± S.E.M. values. Prefrontal cortex, PFC; dorsal striatum, DS; hippocampus, Hipp; cerebellum, CB. White to purple transitions (color key; pg/mL) indicate, respectively, low to high average cytokine or chemokine or VEGF concentration. Data are represented as mean. n = 4 control female, n = 2–3 control male. * *p* < 0.05 vs. all other samples within each sex, # *p* < 0.05 vs. the opposite sex by post-hoc tests.

Differences in the amount of pro-inflammatory and anti-inflammatory cytokines and chemokines were determined between plasma and several brain regions, including the PFC, dorsal striatum, hippocampus and cerebellum in healthy control rats (Figure 2). Two-way ANOVA with sex and plasma/brain regions as independent variables and cytokine/chemokine/VEGF as dependent variables revealed significant effects for each of the markers investigated. IFN-γ: main effect of regions (F4, 21 = 101.8, *p* < 0.001) without an interaction or main effect of sex. Pairwise comparisons revealed higher levels in plasma compared with brain regions in both sexes. TNFα: interaction (F4, 21 = 9.4, *p* = 0.002) main effect of regions (F4, 21 = 9.3, *p* = 0.002) and main effect of sex (F1, 21 = 6.1, *p* = 0.02). Posthoc comparisons revealed higher levels in PFC compared with other regions and plasma in the males, and higher levels in the males in the PFC compared to females. IL-1β: main effect of regions (F4, 21 = 21.3, *p* < 0.001) without an interaction or main effect of sex. Pairwise comparisons revealed higher levels in PFC compared with brain regions and plasma in both sexes. IL-6: interaction (F4, 21 = 8.7, *p* = 0.003), main effect of regions (F4, 21 = 31, *p* < 0.001) without main effect of sex. Post-hoc comparisons revealed higher levels in plasma compared with other regions in females, higher levels in PFC compared with other regions in males, higher levels in the males in the PFC compared to females, and higher levels in the females in the hippocampus compared to males. IL-4: interaction (F4, 21 = 4.7, *p* = 0.007) main effect of regions (F4, 21 = 152.2, *p* = 0.002) without main effect of sex. Post-hoc comparisons revealed higher levels in plasma compared with other regions in both sexes, and higher levels in the males in the PFC compared to females. IL-5: interaction (F4, 21 = 3.2, *p* = 0.03), main effect of regions (F4, 21 = 30.1, *p* < 0.001) without main effect of sex. Post-hoc comparisons revealed lower levels in plasma compared with other regions in both sexes, and higher levels in the males in the PFC compared to females. IL-10: main effect of regions (F4, 21 = 316, *p* < 0.001) without an interaction or main effect of sex. Pairwise comparisons revealed higher levels in plasma compared with brain regions in both sexes. IL-13: main effect of regions (F4, 21 = 79.7, *p* < 0.001) without an interaction or main effect of sex. Pairwise comparisons revealed higher levels in plasma compared with brain regions in both sexes. 

CXCL1: main effect of regions (F4, 21 = 91.8, *p* < 0.001) without an interaction or main effect of sex. Pairwise comparisons revealed higher levels in plasma compared with brain regions and higher levels in plasma in females compared with males. VEGF: interaction (F4, 21 = 20.5, *p* < 0.001), main effect of regions (F4, 21 = 259.1, *p* < 0.001), and main effect of sex (F1, 21 = 9.2, *p* = 0.006). Post-hoc comparisons revealed higher levels in PFC compared with other regions and plasma in the males, and higher levels in the males in the PFC compared to females.

### 3.3. LPS Enhances Peripheral Levels of Cytokines: Sex Specific Effects

**Figure 3:**
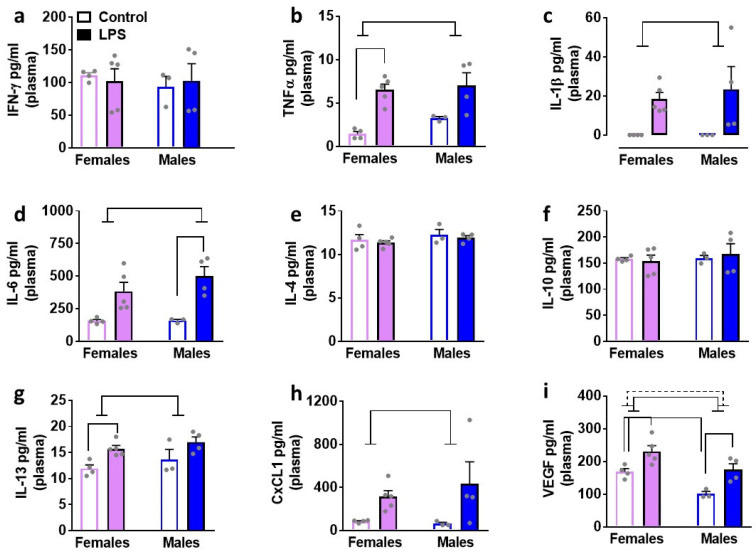
Cytokines, chemokines, and VEGF were measured in the plasma of female and male rats that were exposed to LPS or saline injections. control female, n = 5 LPS female, n = 3 control male, n = 4 LPS male. Pro-inflammatory cytokines are indicated in panels (**a**–**d**). Anti-inflammatory cytokines are indicated in panels (**e**–**g**). Chemokine is indicated in panel (**h**) and angiogenesis marker is indicated in panel (**i**). Main effect of LPS (*p* < 0.05) is indicated by solid line with flat arrowheads and main effect of sex (*p* < 0.05) is indicated by dashed line with flat arrowhead. Differences between saline and LPS within each sex and between sexes (*p* < 0.05) are indicated by separate solid lines.

We determined the effects of LPS on inflammatory cytokines and VEGF in plasma isolated from male and female rats (Figure 3). Two-way ANOVA was performed with sex and LPS as independent variables, and markers as the dependent variable. We report statistical analysis for each marker. TNFα: main effect of LPS (F1, 12 = 26.02, *p* = 0.003) without an interaction or main effect of sex. Pairwise comparisons revealed higher levels after LPS treatment in females compared to saline condition. IL-1β: main effect of LPS (F1, 12 = 10.5, *p* = 0.007) without an interaction or main effect of sex. Pairwise comparisons did not detect any differences. IL-6: main effect of LPS (F1, 12 = 23.5, *p* = 0.004) without an interaction or main effect of sex. Pairwise comparisons revealed higher levels after LPS treatment in males compared to saline condition. IL-13: main effect of LPS (F1, 12 = 12.32, *p* = 0.004) without an interaction or main effect of sex. Pairwise comparisons revealed higher levels after LPS treatment in females compared to saline condition. CXCL1: main effect of LPS (F1, 12 = 7.1, *p* = 0.02) without an interaction or main effect of sex. Pairwise comparisons did not detect any differences. VEGF: main effect of LPS (F1, 12 = 19.4, *p* = 0.009) and main effect of sex (F1, 12 = 15.8, *p* = 0.001) without an interaction. Pairwise comparisons revealed higher levels of VEGF in LPS-treated male and female rats compared with saline-treated controls. Pairwise comparisons also revealed higher levels of VEGF in saline-treated females compared with males. IFN-γ, IL-4, and IL-10 levels were unaltered.

### 3.4. LPS Enhances Specific Cytokines in the Brain Tissue Homogenates: Sex Specific Effects

**Figure 4:**
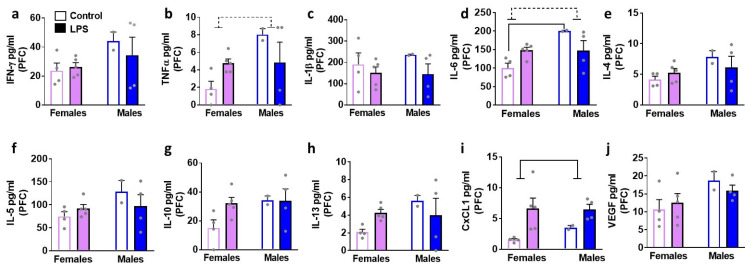
Cytokines, chemokines, and VEGF were measured in the prefrontal cortex (PFC) of female and male rats that were exposed to saline or LPS challenges. Pro-inflammatory cytokines are indicated in panels (**a**–**d**). Anti-inflammatory cytokines are indicated in panels (**e**–**h**). Chemokine is indicated in panel (**i**) and angiogenesis marker is indicated in panel (**j**). Data are represented as mean ± S.E.M. n = 4 control female, n = 5 LPS female, n = 2 control male, n = 4 LPS male. Main effect of LPS (*p* < 0.05) is indicated by solid line with flat arrowheads, and main effect of sex (*p* < 0.05) is indicated by dashed line with flat arrowhead. Differences between saline and LPS within each sex and between sexes (*p* < 0.05) are indicated by separate solid lines.

**Figure 5:**
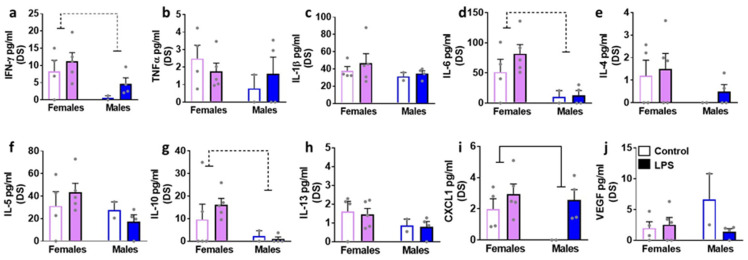
Cytokines, chemokines, and VEGF were measured in the dorsal striatum (DS) of female and male rats that were exposed to saline or LPS challenges. Pro-inflammatory cytokines are indicated in panels (**a**–**d**). Anti-inflammatory cytokines are indicated in panels (**e**–**h**). Chemokine is indicated in panel (**i**) and angiogenesis marker is indicated in panel (**j**). Data are represented as mean ± S.E.M. n = 4 control female, n = 5 LPS female, n = 2 control male, n = 4 LPS male. Main effect of LPS (*p* < 0.05) is indicated by solid line with flat arrowheads, and main effect of sex (*p* < 0.05) is indicated by dashed line with flat arrowhead.

**Figure 6:**
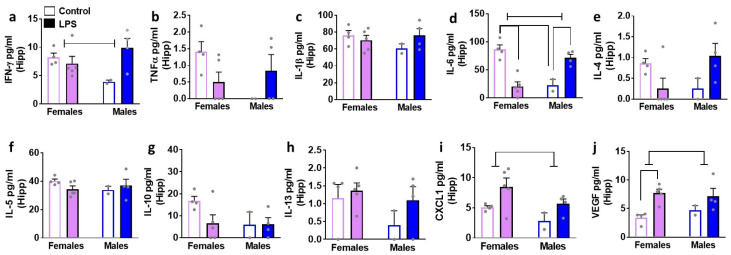
Cytokines, chemokines, and VEGF were measured in the hippocampus (Hipp) of female and male rats that were exposed to saline or LPS challenges. Pro-inflammatory cytokines are indicated in panels (**a**–**d**). Anti-inflammatory cytokines are indicated in panels (**e**–**h**). Chemokine is indicated in panel (**i**) and angiogenesis marker is indicated in panel (**j**). Data are represented as mean ± S.E.M. n = 4 control female, n = 5 LPS female, n = 2 control male, n = 4 LPS male. Interaction (*p* < 0.05) is indicated by a solid straight line with arrowheads, main effect of LPS (*p* < 0.05) is indicated by solid line with flat arrowheads, and main effect of sex (*p* < 0.05) is indicated by dashed line with flat arrowhead. Differences between saline and LPS within each sex and between sexes (*p* < 0.05) are indicated by separate solid lines.

**Figure 7:**
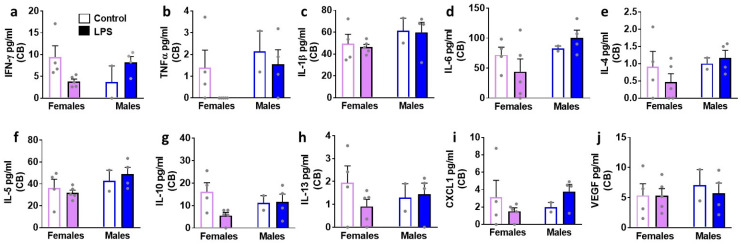
Cytokines, chemokines, and VEGF were measured in the cerebellum (CB) of female and male rats that were exposed to saline or LPS challenges. Pro-inflammatory cytokines are indicated in panels (**a**–**d**). Anti-inflammatory cytokines are indicated in panels (**e**–**h**). Chemokine is indicated in panel (**i**) and angiogenesis marker is indicated in panel (**j**). Data are represented as mean ± S.E.M. n = 4 control female, n = 5 LPS female, n = 2 control male, n = 4 LPS male. No significant differences were seen in any of the markers studied.

We next determined the effects of LPS on inflammatory cytokines and VEGF in brain tissue homogenates isolated in male and female rats (Figure 4, Figure 5, Figure 6 and Figure 7). We list statistically significant data revealed in each brain region.

In the PFC, LPS enhanced levels of CXCL1 in both sexes (main effect of LPS (F1, 11 = 8.8, *p* = 0.01)). All the other markers were unaltered. In the DS, LPS enhanced levels of CXCL1 in both sexes (main effect of LPS (F1, 11 = 6.0, *p* = 0.03)). All the other markers were unaltered. Unlike the PFC and DS, LPS produced significant increases in IL-6, CXCL1, and VEGF in the hippocampus. Details of the statistical analysis include–IL-6: interaction (F1, 11 = 42.6, *p* < 0.001), with post-hoc analysis indicating LPS inducing higher levels in males and lower levels in females. CXCL1: main effect of LPS (F1, 11 = 6.4, *p* = 0.02); VEGF main effect of LPS (F1, 11 = 11.7, *p* = 0.005), with pairwise comparisons revealing higher levels of VEGF in LPS females compared to saline-treated controls. LPS did not produce any significant change in the cerebellum.

### 3.5. LPS Produces Sexually Dimorphic Effects on the Expression of Proteins Essential for Blood-Brain Barrier Integrity

**Figure 8:**
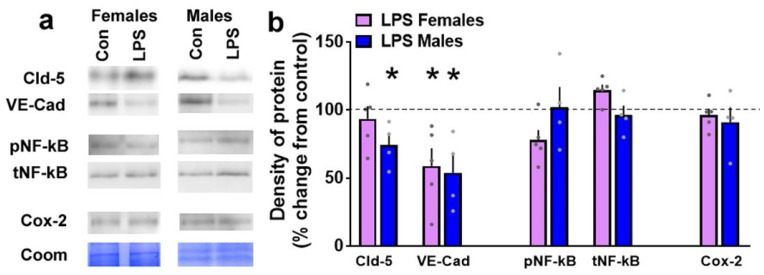
Western blotting analysis of tight junction proteins and neuroinflammatory markers in the prefrontal cortex (PFC) of female and male rats that were exposed to saline or LPS challenges. Representative immunoblots with corresponding Coomassie (Coom) blot is indicated in (**a**). Coom band 150 kDa was used for female samples and band 250/150 kDa was used for male samples. Claudin-5, Cld-5; VE-Cadherin, VE-Cad; phospho NF-kB, pNF-kB; total NF-kB, tNF-kB; control, con. (**b**) Quantitative data represented as mean ± S.E.M. of percent change in density of protein from respective controls (indicated as dashed line). n = 4 control female, n = 5 LPS female, n = 3 control male, n = 4 LPS male. * *p* ≤ 0.05 between saline and LPS within each sex.

We also determined whether LPS altered the expression of proteins critical for BBB integrity in the PFC, as this brain region expressed higher levels of pro-inflammatory cytokines compared with other brain regions (Figure 2). Western blotting revealed that LPS significantly reduced expression of claudin-5 in males (*p* = 0.04) in the PFC and this was not seen in the female rats (Figure 8). LPS reduced levels of VE-cadherin in the PFC in both male (*p* = 0.02) and female rats (*p* = 0.05). LPS did not alter activity and expression of NF-kB or Cox-2 in the PFC.

## 4. Discussion

In this study, we first investigated sex differences in the expression of cytokines, chemokines, and VEGF in plasma and brain tissue homogenates from control healthy rats. 

Our report demonstrates that females have higher baseline levels of chemokines and VEGF in the plasma compared to males. We also show that males have higher baseline expression of cytokines in the brain, specifically in the PFC compared to females (Figure 2). Second, in control healthy rats, within each sex, we examined whether plasma and the brain tissue have differential expression of cytokines, chemokines, and VEGF. Our results indicate that most of the cytokines, chemokines, and VEGF are expressed at higher levels in the plasma compared to brain tissue in both sexes (Figure 2). Notably, our findings reveal higher expression of certain cytokines (IL-1β, IL-5) in the PFC compared to the plasma and other brain regions in both sexes. These findings are, in general, in support of the studies that have indicated higher baseline peripheral immune activity compared to brain tissue homogenates [14,22], higher peripheral immune activity in female rodents compared to males, and add additional information on the higher baseline immune activity in the brains of male rodents compared to females [8,9]. Lastly, we also explored the effects of LPS on plasma and brain tissue homogenates in both sexes (Figure 3, Figure 4, Figure 5, Figure 6 and Figure 7). Contradictory to our hypothesis, our findings reveal that 24 h after two consecutive intraperitoneal LPS challenges, a strong peripheral upregulation of pro-inflammatory cytokines, chemokines and VEGF, and moderate central upregulation of chemokines, particularly in the PFC and hippocampus in both sexes is observed. Additional analysis show that at the 24 h time point after LPS challenges, we see reduced BBB integrity in the PFC, an effect that was more robust in the males compared to the females (Figure 8). Taken together, our findings reveal that 24 h after low to moderate dose LPS challenge, females and males demonstrate robust innate immune response peripherally and mild immune responses centrally without observable sex differences, and moderate BBB dysfunction in the PFC, which was more pronounced in male rats [8,11,22].

LPS is an endotoxin expressed in the outer wall of Gram-negative bacteria. It is recognized by the mammalian immune system as a pathogen-associated molecular pattern (PAMP; [23]). Mechanistically, LPS induces immune responses by signaling via the toll-like receptor 4 (TLR4) and the adapter protein CD14 to enhance nuclear translocation of NF-kB, which leads to an upregulation in the expression of several pro-inflammatory cytokines [24]. Concomitantly, LPS triggers the activation of microglia and the subsequent increases in the expression of cytokines, and it is believed that this is a delayed response that is assisted by injury to the BBB [25]. These immune responses induced by LPS peripherally and centrally occur in parallel with behavioral abnormalities collectively referred to as sickness behavior [26]. Based on several studies, in rat and mouse models, it has been conceptualized that peripheral upregulation of pro-inflammatory cytokines such as IL-1β and TNFα drive the sickness behavior, whereas anti-inflammatory cytokines, such as IL-10, hinder sickness behavior by effecting LPS-induced pro-inflammatory cytokines [27]. Our findings support a prior report in male mice [23] and show that LPS-induced sickness behavior was evident in both sexes, visualized as a significant reduction in body weight 24 h after two consecutive intraperitoneal injections of the drug. We add to the published findings in mice on increased expression of the pro-inflammatory cytokines TNFα, IL-1β, IL-6 in the periphery 4–7 h post-LPS treatment in both sexes [6,9,11,28], to show that this increase is evident in rats 24 h after LPS exposure. Increases in pro-inflammatory cytokines were coupled with higher circulating levels of IL-13, a response that was higher in females 24 h after LPS exposure. This suggests that LPS also regulates aspects of allergic inflammation, perhaps via regulating the responses of T-lymphocytes [29]. We also demonstrate increases in the expression of the chemokine CXCL-1 in the periphery, indicating an antimicrobial response likely contributing to inflammatory and repair responses [30]. In addition to quantifying the levels of cytokines and chemokines in the plasma, we explored whether LPS treatment altered levels of circulating VEGF. This is because in addition to promoting endothelial permeability and proliferation, VEGF has been implicated in pro-inflammatory responses, pro-coagulant activity, and mediating the sepsis phenotype. Notably, these responses occur via NF-kB signaling, suggesting pro-inflammatory signaling under angiogenic insult [31,32,33]. Our findings support the previously published finding of very high dose of LPS (18 mg/kg)-induced enhanced circulating VEGF levels in male mice at 24 h post injection [32] and extend these findings to male and female rats that received low to moderate dose (1 mg/kg) of LPS. 

It is important to note that the kinetics of LPS response in cytokines are different in the periphery and brain after a single LPS challenge. The peak at which LPS induces peripheral inflammatory cytokine secretion generally precedes a central response [14]. However, it appears that the chemokines have similar kinetics in the brain and periphery after a single LPS challenge [14]. Interestingly, the kinetics of LPS response seem similar in brain and periphery after multiple injections of LPS, and this regimen produced higher brain responses compared with a single-injection regimen [14]. However, the effect of multiple LPS injections on brain cytokines and chemokines beyond 4–7 h after the final LPS challenge has been rarely explored. Previous research demonstrates that in adult rodents, a high to very-high dose of LPS (3–30 mg/kg) enhances brain cytokine and chemokine levels, albeit to a lesser extent compared with peripheral levels [11,14,22]. Our findings with a low to moderate dose of LPS (1 mg/kg) show that cytokines were mostly unaltered 24 h after the final LPS challenge in the brain homogenates. Notably, within the brain, LPS elevated cortical, striatal, and hippocampal levels of the chemokine CXCL1, albeit to a lesser extent than in the periphery in both sexes. The cerebellum was unaffected. These findings demonstrate that the dose of LPS and time point after LPS challenge play an important role in directing the central effects of LPS and could be related to the fact that higher multiple doses of LPS are required to produce significant disruption in BBB integrity [11]. Given that cortical, striatal, and hippocampal regions were significantly affected even after low to moderate dose of LPS, and that the cerebellum was spared, it is tempting to speculate that the cognitive deficits and negative affective behaviors seen after LPS could be associated with immune responses in these brain regions during adulthood [9]. Nevertheless, the central effects in CXCL1, in conjunction with the peripheral increases of cytokines and CXCL1 after low to moderate dose of LPS, could contribute to and drive the sickness behavior post LPS [27].

Neonatal LPS exposure produces profound alterations in microglial morphology in the PFC in middle-aged male and female rats, indicating an activated state, and an effect that was evident months after challenge. In adult male rats, studies show that 24 h after exposure to a low to moderate dose, LPS increases microglial activity in the PFC [19,34], a brain region that plays a significant role in cognitive performance [35,36]. Notably, activated microglia, specifically in the cortex, associated with inflammatory insult during early postnatal development in rodents, is influenced by CXCL1 signaling [37], suggesting a role for brain chemokines in microglial activation. While the mechanism by which peripheral LPS can alter neuroimmune signaling is still an area of active study, intraperitoneal LPS could activate brain microglial cells via vagal nerve activity, transport of pro-inflammatory products like cytokines across the BBB, or signaling of epithelial cells intrinsic in the barrier itself [38,39]. Since our findings reveal higher expression of a few cytokines in the PFC compared to plasma and other brain regions at basal healthy state, we further explored the effect of LPS on neuroinflammatory factors and proteins associated with BBB integrity in the PFC. The integrity of the BBB is maintained by tight junctions and adherens junctions, which are located closest to the apical membrane of endothelial cells. These junctions prevent the diffusion of proteins between luminal and abluminal membrane compartments of the endothelial cells. Claudin-5 in an integral component of the tight junction, and VE-cadherin in integral component of the adherens junction, and their expression is directly responsible for determining the permeability of the tight junctions [40,41]. Our study extends previous reports and adds to the findings to demonstrate that LPS challenge increased chemokines in the PFC, and in parallel, reduced proteins associated with BBB integrity (tight junction protein claudin-5 and adheren junction protein VE-cadherin) in both sexes. Of interest is the fact that systemic LPS challenge produces profound transcriptional activation of the gene encoding Cox-2 along the brain vascular cells via NF-kB signaling [42,43]. Since we demonstrate reduced expression of tight junction proteins, such as cadherins in the PFC post LPS challenge, it is likely that LPS challenge-induced circulating pro-inflammatory cytokines and leaky BBB could stimulate Cox-2 production via transcriptional activation of NF-kB signaling in the PFC. Western blotting analysis did not reveal any upregulation of Cox-2 or activation of NF-kB in the PFC, suggesting that reduced expression of tight junction proteins did not occur coincidentally with the induction of Cox-2 and activation of NF-kB. A potential limitation in the interpretation of these results is that analysis of these markers at additional time points after LPS challenge is required to confirm this effect. In addition, it needs to be determined whether the changes observed in the PFC with these markers are specific to this brain region or can be generalized to other regions in the brain. 

In conclusion, while we attempted to discuss our findings in relation to the published work, the vast literature on LPS-induced peripheral and neuroimmune responses mostly based on studies conducted in mouse models, the several doses of LPS used, and various time points of euthanasia post-LPS challenge make it challenging to directly relate our findings to the available reports. Overall, this study demonstrates that exposure to an environmentally relevant dose of endotoxin during adulthood can lead to disruption in circulating pro-inflammatory cytokines and neuroimmune activity in male and female rats. Coincidentally, LPS challenge also disrupted levels of chemokines in the PFC, dorsal striatum and hippocampus, and tight junction proteins in the PFC in both sexes. Importantly, the sex-specific differences in the expression of pro-inflammatory cytokines observed in both basal and LPS-challenged states in the plasma and brain tissue homogenates emphasizes the importance of assessing the effects of any possible immunotoxin on both male and female rodents.

## Data Availability

Data will be made available upon request to the corresponding author.

## References

[1] Dantzer R., O’Connor J.C., Freund G.G., Johnson R.W., Kelley K.W. (2008). From inflammation to sickness and depression: When the immune system subjugates the brain. Nat. Rev. Neurosci..

[2] Capuron L., Miller A.H. (2004). Cytokines and psychopathology: Lessons from interferon-alpha. Biol. Psychiatry.

[3] Karshikoff B., Lekander M., Soop A., Lindstedt F., Ingvar M., Kosek E., Olgart Höglund C., Axelsson J. (2015). Modality and sex differences in pain sensitivity during human endotoxemia. Brain Behav. Immun..

[4] Engler H., Benson S., Wegner A., Spreitzer I., Schedlowski M., Elsenbruch S. (2016). Men and women differ in inflammatory and neuroendocrine responses to endotoxin but not in the severity of sickness symptoms. Brain Behav. Immun..

[5] Hopwood N., Maswanganyi T., Harden L.M. (2009). Comparison of anorexia, lethargy, and fever induced by bacterial and viral mimetics in rats. Can. J. Physiol. Pharmacol..

[6] Wendeln A.C., Degenhardt K., Kaurani L., Gertig M., Ulas T., Jain G., Wagner J., Häsler L.M., Wild K., Skodras A. (2018). Innate immune memory in the brain shapes neurological disease hallmarks. Nature.

[7] Camara M.L., Corrigan F., Jaehne E.J., Jawahar M.C., Anscomb H., Baune B.T. (2015). Effects of centrally administered etanercept on behavior, microglia, and astrocytes in mice following a peripheral immune challenge. Neuropsychopharmacology.

[8] Berkiks I., Mesfioui A., Ouichou A., Nakache R., Ajonijebu D.C., El Hessni A. (2019). Affective Behavior Shows Sex Differences in Mid-adulthood Rats Following Postnatal Immune Stimulation. Neuroscience.

[9] Dockman R.L., Carpenter J.M., Diaz A.N., Benbow R.A., Filipov N.M. (2022). Sex differences in behavior, response to LPS, and glucose homeostasis in middle-aged mice. Behav. Brain Res..

[10] Liberman D.A., Walker K.A., Gore A.C., Bell M.R. (2020). Sex-specific effects of developmental exposure to polychlorinated biphenyls on neuroimmune and dopaminergic endpoints in adolescent rats. Neurotoxicol. Teratol..

[11] Erickson M.A., Liang W.S., Fernandez E.G., Bullock K.M., Thysell J.A., Banks W.A. (2018). Genetics and sex influence peripheral and central innate immune responses and blood-brain barrier integrity. PLoS ONE.

[12] Dantzer R. (2018). Neuroimmune Interactions: From the Brain to the Immune System and Vice Versa. Physiol. Rev..

[13] Thomson C.A., McColl A., Graham G.J., Cavanagh J. (2020). Sustained exposure to systemic endotoxin triggers chemokine induction in the brain followed by a rapid influx of leukocytes. J. Neuroinflamm..

[14] Erickson M.A., Banks W.A. (2011). Cytokine and chemokine responses in serum and brain after single and repeated injections of lipopolysaccharide: Multiplex quantification with path analysis. Brain Behav. Immun..

[15] Bouman A., Heineman M.J., Faas M.M. (2005). Sex hormones and the immune response in humans. Hum. Reprod. Update.

[16] Pennell L.M., Galligan C.L., Fish E.N. (2012). Sex affects immunity. J. Autoimmun..

[17] Martin E.I., Ressler K.J., Binder E., Nemeroff C.B. (2010). The neurobiology of anxiety disorders: Brain imaging, genetics, and psychoneuroendocrinology. Clin. Lab. Med..

[18] Lago T., Davis A., Grillon C., Ernst M. (2017). Striatum on the anxiety map: Small detours into adolescence. Brain Res..

[19] Siemsen B.M., Landin J.D., McFaddin J.A., Hooker K.N., Chandler L.J., Scofield M.D. (2020). Chronic intermittent ethanol and lipopolysaccharide exposure differentially alter Iba1-derived microglia morphology in the prelimbic cortex and nucleus accumbens core of male Long-Evans rats. J. Neurosci. Res..

[20] Welinder C., Ekblad L. (2011). Coomassie staining as loading control in Western blot analysis. J. Proteome Res..

[21] Thacker J.S., Yeung D.H., Staines W.R., Mielke J.G. (2016). Total protein or high-abundance protein: Which offers the best loading control for Western blotting?. Anal. Biochem..

[22] Abramova A.Y., Pertsov S.S., Kozlov A.Y., Nikenina E.V., Kalinichenko L.S., Dudnik E.N., Alekseeva I.V. (2013). Cytokine levels in rat blood and brain structures after administration of lipopolysaccharide. Bull. Exp. Biol. Med..

[23] Spulber S., Edoff K., Hong L., Morisawa S., Shirahata S., Ceccatelli S. (2012). Molecular hydrogen reduces LPS-induced neuroinflammation and promotes recovery from sickness behaviour in mice. PLoS ONE.

[24] Zhang G., Ghosh S. (2000). Molecular mechanisms of NF-kappaB activation induced by bacterial lipopolysaccharide through Toll-like receptors. J. Endotoxin. Res..

[25] Rivest S. (2003). Molecular insights on the cerebral innate immune system. Brain Behav. Immun..

[26] Dantzer R., Kelley K.W. (2007). Twenty years of research on cytokine-induced sickness behavior. Brain Behav. Immun..

[27] Dantzer R. (2004). Cytokine-induced sickness behaviour: A neuroimmune response to activation of innate immunity. Eur. J. Pharmacol..

[28] Everhardt Queen A., Moerdyk-Schauwecker M., McKee L.M., Leamy L.J., Huet Y.M. (2016). Differential Expression of Inflammatory Cytokines and Stress Genes in Male and Female Mice in Response to a Lipopolysaccharide Challenge. PLoS ONE.

[29] Marone G., Granata F., Pucino V., Pecoraro A., Heffler E., Loffredo S., Scadding G.W., Varricchi G. (2019). The Intriguing Role of Interleukin 13 in the Pathophysiology of Asthma. Front. Pharmacol..

[30] Jin L., Batra S., Douda D.N., Palaniyar N., Jeyaseelan S. (2014). CXCL1 contributes to host defense in polymicrobial sepsis via modulating T cell and neutrophil functions. J. Immunol..

[31] Kim I., Moon S.O., Kim S.H., Kim H.J., Koh Y.S., Koh G.Y. (2001). Vascular endothelial growth factor expression of intercellular adhesion molecule 1 (ICAM-1), vascular cell adhesion molecule 1 (VCAM-1), and E-selectin through nuclear factor-kappa B activation in endothelial cells. J. Biol. Chem..

[32] Yano K., Liaw P.C., Mullington J.M., Shih S.C., Okada H., Bodyak N., Kang P.M., Toltl L., Belikoff B., Buras J. (2006). Vascular endothelial growth factor is an important determinant of sepsis morbidity and mortality. J. Exp. Med..

[33] Reinders M.E., Sho M., Izawa A., Wang P., Mukhopadhyay D., Koss K.E., Geehan C.S., Luster A.D., Sayegh M.H., Briscoe D.M. (2003). Proinflammatory functions of vascular endothelial growth factor in alloimmunity. J. Clin. Investig..

[34] Kongsui R., Johnson S.J., Graham B.A., Nilsson M., Walker F.R. (2015). A combined cumulative threshold spectra and digital reconstruction analysis reveal structural alterations of microglia within the prefrontal cortex following low-dose LPS administration. Neuroscience.

[35] Anderson M.C., Floresco S.B. (2022). Prefrontal-hippocampal interactions supporting the extinction of emotional memories: The retrieval stopping model. Neuropsychopharmacology.

[36] Jones D.T., Graff-Radford J. (2021). Executive Dysfunction and the Prefrontal Cortex. Continuum (Minneap Minn).

[37] Serdar M., Kempe K., Herrmann R., Picard D., Remke M., Herz J., Bendix I., Felderhoff-Müser U., Sabir H. (2020). Involvement of CXCL1/CXCR2 During Microglia Activation Following Inflammation-Sensitized Hypoxic-Ischemic Brain Injury in Neonatal Rats. Front. Neurol..

[38] Hoogland I.C.M., Westhoff D., Engelen-Lee J.Y., Melief J., Valls Serón M., Houben-Weerts J., Huitinga I., van Westerloo D.J., van der Poll T., van Gool W.A. (2018). Microglial Activation After Systemic Stimulation With Lipopolysaccharide and Escherichia coli. Front. Cell Neurosci..

[39] Nakano Y., Furube E., Morita S., Wanaka A., Nakashima T., Miyata S. (2015). Astrocytic TLR4 expression and LPS-induced nuclear translocation of STAT3 in the sensory circumventricular organs of adult mouse brain. J. Neuroimmunol..

[40] Greene C., Hanley N., Campbell M. (2019). Claudin-5: Gatekeeper of neurological function. Fluids Barriers CNS.

[41] Li W., Chen Z., Chin I., Chen Z., Dai H. (2018). The Role of VE-cadherin in Blood-brain Barrier Integrity Under Central Nervous System Pathological Conditions. Curr. Neuropharmacol..

[42] Inoue H., Tanabe T. (1998). Transcriptional role of the nuclear factor kappa B site in the induction by lipopolysaccharide and suppression by dexamethasone of cyclooxygenase-2 in U937 cells. Biochem. Biophys. Res. Commun..

[43] Lacroix S., Rivest S. (1998). Effect of acute systemic inflammatory response and cytokines on the transcription of the genes encoding cyclooxygenase enzymes (COX-1 and COX-2) in the rat brain. J. Neurochem..

